# A novel oxidative stress- and ferroptosis-related gene prognostic signature for distinguishing cold and hot tumors in colorectal cancer

**DOI:** 10.3389/fimmu.2022.1043738

**Published:** 2022-10-31

**Authors:** Xu Wang, Yuanmin Xu, Longfei Dai, Zhen Yu, Ming Wang, Shixin Chan, Rui Sun, Qijun Han, Jiajie Chen, Xiaomin Zuo, Zhenglin Wang, Xianyu Hu, Yang Yang, Hu Zhao, Kongwang Hu, Huabing Zhang, Wei Chen

**Affiliations:** ^1^ Department of General Surgery, The First Affiliated Hospital of Anhui Medical University, Hefei, Anhui, China; ^2^ Department of Dermatology, The First Affiliated Hospital of Anhui Medical University, Hefei, Anhui, China; ^3^ Department of Biochemistry and Molecular Biology, Metabolic Disease Research Center, School of Basic Medicine, Anhui Medical University, Hefei, Anhui, China; ^4^ The First Affiliated Chuzhou Hospital of Anhui Medical University, Chuzhou, Anhui, China

**Keywords:** oxidative stress, ferroptosis, colorectal cancer, prognosis, tumor microenvironment, chemotherapy, immunotherapy

## Abstract

Oxidative stress and ferroptosis exhibit crosstalk in many types of human diseases, including malignant tumors. We aimed to develop an oxidative stress- and ferroptosis-related gene (OFRG) prognostic signature to predict the prognosis and therapeutic response in patients with colorectal cancer (CRC). Thirty-four insertion genes between oxidative stress-related genes and ferroptosis-related genes were identified as OFRGs. We then performed bioinformatics analysis of the expression profiles of 34 OFRGs and clinical information of patients obtained from multiple datasets. Patients with CRC were divided into three OFRG clusters, and differentially expressed genes (DEGs) between clusters were identified. OFRG clusters correlated with patient survival and immune cell infiltration. Prognosis-related DEGs in three clusters were used to calculate the risk score, and a prognostic signature was constructed according to the risk score. In this study, patients in the low-risk group had better prognosis, higher immune cell infiltration levels, and better responses to fluorouracil-based chemotherapy and immune checkpoint blockade therapy than high-risk patients; these results were successfully validated with multiple independent datasets. Thus, low-risk CRC could be defined as hot tumors and high-risk CRC could be defined as cold tumors. To further identify potential biomarkers for CRC, the expression levels of five signature genes in CRC and adjacent normal tissues were further verified *via* an *in vitro* experiment. In conclusion, we identified 34 OFRGs and constructed an OFRG-related prognostic signature, which showed excellent performance in predicting survival and therapeutic responses for patients with CRC. This could help to distinguish cold and hot tumors in CRC, and the results might be helpful for precise treatment protocols in clinical practice.

## Introduction

Colorectal cancer (CRC) has become the third most common malignant tumor in males and the second most common in females worldwide, with nearly 19 million new cases and 10 million cancer-related deaths observed in 2020 (1). In addition, it accounts for approximately 10% of all cancer types and is the second most frequent cause of cancer-related death (2, 3). Therefore, CRC has become a global public health challenge owing to its increasing incidence, mortality, and requirement for medical service utilization (4). A certain proportion of patients are diagnosed with advanced CRC, and metastases are observed in approximately 20% of diagnosed CRC cases (5). Clinical outcomes for CRC patients have improved markedly over the past few decades, which is credited to advances in surgical approaches, anti-cancer drugs, and other more effective therapeutic approaches. In addition to traditional chemotherapy, immunotherapy has been used for cancer treatment in recent years, and patients with metastatic CRC with high microsatellite instability or deficient mismatch repair can significantly benefit from immune checkpoint blockade therapy (6). However, majority of patients with CRC cannot benefit considerably from immunotherapy. To solve this problem, more biomarkers have been identified, such as tumor burden mutation (TMB), microsatellite instability, and neoantigen load (NAL); nevertheless, the predictive ability of these biomarkers is limited because they are associated with small proportions of the population or moderate predicting efficiency. In addition, although some tumor markers and the clinical stage can be used to predict patient prognosis in CRC, these variables cannot be used to efficiently predict chemotherapy or immunotherapy benefits. Thus, developing a novel approach to distinguish cold and hot tumors is vital for the individualized treatment of patients with CRC.

Oxidative stress is defined as a relative excess of reactive oxygen species (ROS) when compared with antioxidant levels, and it has been proven to be associated with various types of human diseases. Aberrant redox homeostasis can be observed in cancer cells; although ROS can promote tumor growth, high ROS levels also have toxic effects on malignant tumors (7). The excessive proliferation of tumor cells is often accompanied by enhanced ROS production; however, tumor cells can grow under conditions in which this oxidative load pushes the redox balance away from a reduced state. Moreover, tumor cells optimize ROS-driven proliferation by increasing their antioxidant status, while avoiding ROS thresholds that trigger cellular senescence, apoptosis, or ferroptosis to achieve this (8, 9). In recent years, many researchers have focused on increasing ROS levels in cancer cells to induce ROS-mediated cell death, which is defined as oxidation therapy (10–12).

Resistance to cell death has been proven to be one of the basic cancer hallmarks (13). Apoptosis was once considered the only form of programmed cell death (PCD); however, with a broader understanding of such processes, more new forms of PCD have been identified, including ferroptosis (14), pyroptosis (15), necroptosis (16), and cuproptosis (17). Ferroptosis is a new type of PCD that is iron-dependent, and it differs from apoptosis, necroptosis, and autophagy. The ferroptosis process is often accompanied by the accumulation of large amounts of iron ions, occurrence of lipid peroxidation, and increase in ROS. In terms of cell structural changes, mitochondria appear smaller than those in normal cells, and the mitochondrial membrane shrinks, while the mitochondrial cristae decrease or disappear, and the outer membrane breaks; nonetheless, morphological changes in the nucleus are not obvious (14, 18, 19). Many recent studies have elaborated on the role of ferroptosis in cancer (20–22). As such, strategies to control ferroptosis induction could effectively inhibit tumor development, even in tumors that show resistance to chemotherapy (22, 23).

Since crosstalk between oxidative stress and ferroptosis has been found in many human diseases, exploring the role of oxidative stress and ferroptosis-related genes (OFRGs) in CRC might help to develop new treatment strategies. In this study, OFRGs were identified and their expression levels, genetic alterations, and prognostic value in CRC were evaluated. Patients were classified into three OFRG clusters, and prognosis-related DEGs between OFRG clusters were used to construct the prognostic signature, which showed satisfactory efficiency in predicting patient survival, the tumor immune microenvironment (TME), chemotherapy effects, and immunotherapy benefits. These results were successfully validated based on multiple independent cohorts from different public datasets. Low-risk patients had a significantly longer survival time than high-risk patients; they also showed higher therapeutic sensitivity after receiving fluorouracil-based chemotherapy or immune checkpoint blockade therapy, indicating that our signature could help distinguish cold and hot tumors in CRC, which might provide a reference for precise mediation in clinical practice.

## Material and methods

### Collection and processing of transcriptional and clinical data

Transcriptional and clinical information of patients in 13 independent public datasets was retrieved from The Cancer Genome Atlas (TCGA, https://portal.gdc.cancer.gov), Gene Expression Omnibus (GEO, https://www.ncbi.nlm.nih.gov/geo/, ID: GSE39582, GSE17536, GSE17537, GSE29621, GSE38832, GSE19860, GSE45404, GSE62080, and GSE78820), iMvigor210 (http://research-pub.gene.com/IMvigor210CoreBiologies), and Tumor Immune Dysfunction and Exclusion (TIDE) website (https://tide.dfci.harvard.edu/, ID: PRJEB23709 and PRJEB25780). Among these datasets, six datasets (TCGA-CRC, GSE39582, GSE17536, GSE17537, GSE29621, and GSE38832) containing complete follow-up information were used for constructing our prognostic signature and verifying its efficiency in predicting the survival of patients with CRC. For drug-related datasets, three CRC datasets (GSE19860, GSE45404, and GSE62080) comprising patients treated with fluorouracil-based chemotherapy (FOLFOX or FOLFIRI) were used in our study. In addition, four immunotherapy-related datasets (GSE78820, iMvigor210, PRJEB23709 and PRJEB25780) for melanoma, urothelial, and metastatic gastric cancers, comprising treatment with *PD-1*, *PD-L1*, or *CTLA-4* blockade therapy, were used to evaluate the performance of our signature in predicting immunotherapy benefits. Fragments per kilobase million data from TCGA-CRC cohort were transformed into transcripts per million using R studio software (version 1.4.1106). All CRC datasets from the GEO database were downloaded from the GPL570 platform (Affymetrix Human Genome U133 Plus 2.0 Array). TCGA-CRC data and GSE39582 data were combined and used as the training group, and batch effects were removed using the *ComBat* algorithm. Expression profiles were normalized and log2 transformed using the *sva* R package. Patients with missing overall survival (OS) or clinical information were excluded from our study.

### Genetic and transcriptional alterations to OFRGs in CRC

Oxidative stress-related genes (ORGs) were retrieved from the Genecards database (https://www.genecards.org/), the top 200 genes with the highest relevance score were identified as ORGs, and a list of ferroptosis-related genes (FRGs) was downloaded from the FerrDb database (http://www.zhounan.org/ferrdb/current/). Thirty-four insertion genes between ORGs and FRGs were identified as OFRGs. Gene Ontology (GO) and Kyoto Encyclopedia of Genes and Genomes (KEGG) analyses were performed to explore relevant biological functions and pathways in which these OFRGs are involved. Copy number variation frequency and locations of OFRGs in chromosomes were analyzed and presented. The expression levels between normal and CRC tissues were compared and analyzed using the Wilcoxon method with the *limma* package of R software. Prognostic values of OFRGs in patients with CRC were evaluated using KM and univariate Cox regression methods.

### Consensus clustering to identify OFRG clusters

Based on 34 OFRGs, a consensus clustering method was performed to classify patients into distinct OFRG clusters. The classification with the lowest intergroup and highest intragroup correlations were identified by increasing the clustering variable *k.* Principal component analysis (PCA) was used to distinguish three OFRG clusters with the *stats* R package. The OS of patients in different clusters was analyzed using the KM method and log-rank test with *survival* and *survminer* R packages. Clinical characteristics and outcomes of patients in OFRG clusters were compared, and differentially expressed genes (DEGs) were identified with the criteria fold-change >1.5 and *p*-value <0.05. Gene set variation analysis (GSVA) and single-sample gene set enrichment analysis (ssGSEA) were applied to explore immune cell infiltration and immune-related pathways.

### Construction and validation of the OFRGs-related prognostic signature

Univariate Cox regression analysis was performed to identify prognosis-related DEGs (PRDEGs) between OFRG clusters. Least absolute shrinkage and selection operator (LASSO) and stepwise Cox regression analyses were applied to screen genes for constructing the prognostic signature using the *survival*, *survminer*, and *glmnet* R packages. The risk score was calculated based on gene expression levels and corresponding coefficient values. Based on the risk score, patients were divided into high- and low-risk groups, and the survival status and survival times in different risk groups were compared. The receiver operating characteristic (ROC) method was performed to evaluate the efficiency of the risk score in predicting patient survival. Proportion of clinicopathologic factors in high- and low-risk groups was shown in pie charts using Chi-squared test. addition, the results were verified using four independent CRC cohorts from the GEO database. Univariate and multivariate Cox regression analyses were used to determine independent prognostic characteristics of patients with CRC in the training cohort, and a nomogram model was established to predict patient prognosis more accurately based on the results of Cox regression analysis. A calibration graph was generated to show the differences between nomogram-predicted survival rates and actual observed survival rates of patients with CRC.

### Immune cell infiltration differences in high- and low-risk groups

Immune cell infiltration in CRC tissues was quantified using the CIBERSORT algorithm, and the Spearman method was applied to analyze the correlation between risk score and abundance of infiltrating immune cells. The association between signature genes and immune cells was also analyzed. Immune-related scores, including stromal, immune, and ESTIMATE scores, were compared between high- and low-risk groups. Then, pathology slide images were downloaded from TCGA database, and the images were used to show the differences in immune cell infiltration between high- and low-risk patients.

### Relationship between risk score and IC_50_ values of therapeutic drugs

The IC_50_ is the half-maximal inhibitory concentration and represents the concentration of the drug required to achieve 50% inhibition in cell lines. Using the *pRRophetic* R package, IC_50_ values of different therapeutic drugs in high- and low-risk groups were compared using the Wilcoxon signed rank test, including the first line chemotherapeutic drug 5-fluorouracil.

### Efficiency of risk score in predicting patient response to fluorouracil-based adjuvant chemotherapy and bevacizumab

All patients with CRC from GSE19860, GSE45404, and GSE62080 datasets were treated with fluorouracil-based ACT. Among the three datasets, 12 patients in the GSE19860 dataset received additional bevacizumab therapy. Patients were divided into no-response (NR) and response (R) groups according to therapeutic responses. The risk scores of high- and low-risk patients in these three datasets were compared using a Wilcoxon signed rank test, and the proportions of patients in NR and R groups were calculated.

### Immune checkpoints expression, TIDE score, and immune cell proportion score in the high- and low-risk groups

Expression levels of some well-known immune checkpoint genes were compared between high- and low-risk groups using the Wilcoxon signed rank test. TIDE scores were retrieved from the TIDE website, and IPS data were downloaded from The Cancer Immunome Atlas (TCIA, https://tcia.at/). The TIDE score was applied to evaluate the probability of tumor immune escape, with higher TIDE score representing an increased likelihood of immune escape and less benefit from immunotherapy. IPS was used to predict the response to various types of ICI therapy in patients, including *PD-1*/*PD-L1*/*PD-L2*, *CTLA-4*, *CTLA-4*, and *PD-1*/*PD-L1*/*PD-L2* blockade therapy. TIDE score and the IPS were also compared between high- and low-risk groups.

### Evaluating the performance of risk score in predicting immunotherapy benefits

IMvigor210 is a clinical cohort of patients with urothelial carcinoma who received *PD-L1* blockade immunotherapy. GSE78820 contains transcriptional and clinical information of patients with melanoma who received *PD-1* blockade therapy. PRJEB23709 includes tumor biopsies from melanoma patients treated with anti-*PD-1* monotherapy or combined anti-*PD-1* and anti-*CTLA-4* agents. Patients with metastatic gastric cancer with complete follow-up and transcriptional information from the PRJEB25780 cohort were also included in our study. According to responses to immunotherapy, patients were classified into complete response (CR)/partial response (PR) and stable disease (SD)/progressive disease (PD) groups. The risk score between two groups was calculated and compared, and the proportion of CR/PR and SD/PD patients in each cohort was determined.

### *In-vitro* verification of signature genes using quantitative real-time polymerase chain reaction

Ten pairs of CRC and adjacent normal tissues were collected from patients after surgical resection at The First Affiliated Hospital of Anhui Medical University, and the experiments were approved by the Ethics Committee. The samples were stored at -80°C until use. All participants provided signed informed consent. TRIzol reagent (Invitrogen, Carlsbad, CA, USA) was applied to extract total RNA from the collected samples, and the PrimeScript RT kit (Vazyme, Nanjing, China) was used to transcribe extracted RNA into cDNA. The concentrations of all cDNA samples were measured using TB Green Premix Ex Taq II (GenStar, China) with the LightCycler480 System (Applied Biosystems, Waltham, MA, United States). The relative expression levels of signature genes were computed through the 2^-ΔΔCt^ strategy, normalizing to levels of GAPDH. The expression levels between normal and tumor tissues were compared using paired and unpaired t-tests. The primer sequences used for qRT-PCR are presented in **Supplementary Table S1**.

## Results

### Genetic and transcriptional alterations of OFRGs in CRC

**Figure 1:**
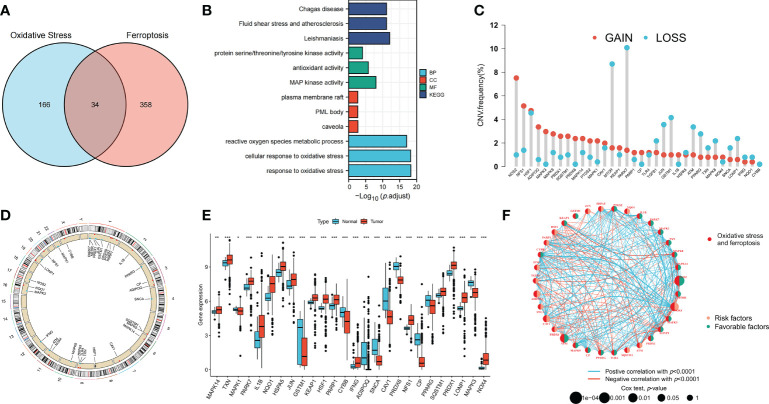
Genetic, transcriptional alterations and functional analyses of OFRGs in CRC. **(A)** Identification of 34 OFRGs. **(B)** GO and KEGG analyses of OFRGs. **(E)** Expression levels of differentially expressed OFRGs between normal and tumor samples. **(C)** Frequencies of CNV gain, loss, and non-CNV among OFRGs. **(D)** Locations of CNV alterations in OFRGs on 23 chromosomes. **(F)** Interactions among OFRGs in CRC. The lines among the genes represent their interactions. Blue and red represent positive and negative correlations. *p < 0.05; **p < 0.01; ***p < 0.001.

The clinical information of patients in all datasets is shown in **Supplementary Table S2**. Thirty-four OFRGs were identified after considering the insertion between ORGs and FRGs (**Figure 1A**; **Supplementary Table S3**). GO and KEGG analyses were performed to explore OFRG-related biological processes (BP), cellular components, molecular functions, and pathways (**Figure 1B**). The results showed that these OFRGs were mainly associated with the following BP: ROS metabolic process, cellular response to oxidative stress, and response to oxidative stress, indicating that OFRGs were closely related to oxidative stress. The copy number variation (CNV) among the OFRGs in CRC was also analyzed (**Figure 1C**). *NOS2*, *NFS1*, *HSF1*, *ADIPOQ*, *MAPK3*, and *MAPK9* showed the most widespread CNV increases, whereas *PARK7*, *MTOR*, *GSTM1*, *JUN*, and *TGFB1* showed CNV decreases. The locations of CNVs in the OFRGs on human chromosomes are presented in **Figure 1D**. The somatic mutation incidence of OFRGs in patients was also calculated (**Supplementary Figure S1**). The expression levels of OFRGs between normal and tumor samples from TCGA database were compared (**Figure 1E**). A network among OFRGs was constructed to show the interactions between the OFRGs and their prognostic significance (**Figure 1F**). KM curves of prognosis-related OFRGs are also shown (**Supplementary Figure S2**).

### Identification of OFRG clusters using consensus clustering

**Figure 2:**
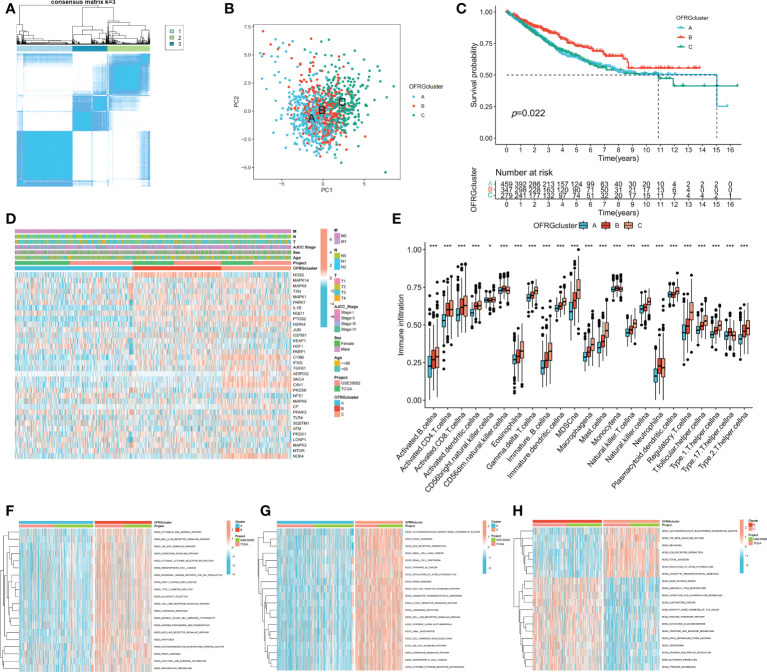
OFRG clusters and clinical characteristics, tumor microenvironment between CRC samples in OFRG clusters. **(A)** Three OFRG clusters were defined using consensus clustering analyses. **(B)** PCA showed the distinction between three OFRG clusters. **(C)** The KM curve revealed significant difference in the survival time between the three clusters (*p* = 0.022); **(D)** Heatmaps showed the relationship between OFRG clusters and clinical features and OFRGs expression in patients with CRC. **(E)** ssGSEA investigated the differences of immune cell infiltration between OFRG clusters. **(F–H)** GSVA showed the enriched pathways between each two OFRG clusters. *p < 0.05, ***p < 0.001.

Patients with CRC were classified into three OFRG clusters using a consensus clustering method based on expression levels of OFRGs (**Figure 2A**). Separation between OFRG clusters was shown using PCA (**Figure 2B**). The KM curve indicated that OFRG cluster B had more favorable outcomes than OFRG clusters A and C (**Figure 2C**). The expression levels of OFRGs and clinical characteristics in OFRG clusters are shown in the heatmap (*p* = 0.022, **Figure 2D**). The results of ssGSEA revealed that cluster C had the highest immune cell infiltration levels, whereas the lowest immune cell infiltration levels were observed in cluster A (**Figure 2E**, *p* < 0.05). In addition, related enriched pathways between each of the two OFRG clusters were compared using the GSVA method (**Figures 2F–H**).

### Construction and validation of the OFRGs-related prognostic signature

**Figure 3:**
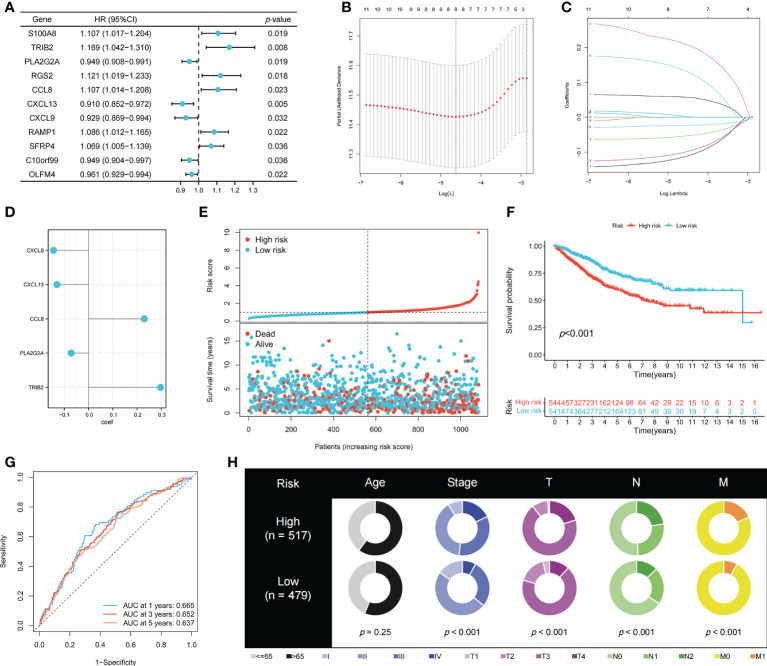
Construction of the prognostic signature. **(A)** PRDEGs were identified using univariate Cox regression analysis. **(B, C)** The LASSO regression analysis and partial likelihood deviance on the prognostic genes. **(D)** The coefficient values of the multivariate Cox regression. **(E)** Risk score and survival outcome of each case. KM **(F)** and ROC **(G)** curves showing the prognostic value in the training cohort. **(H)** Pie charts showing the Chi-squared test of clinicopathologic factors in high- and low-risk groups.

**Figure 4:**
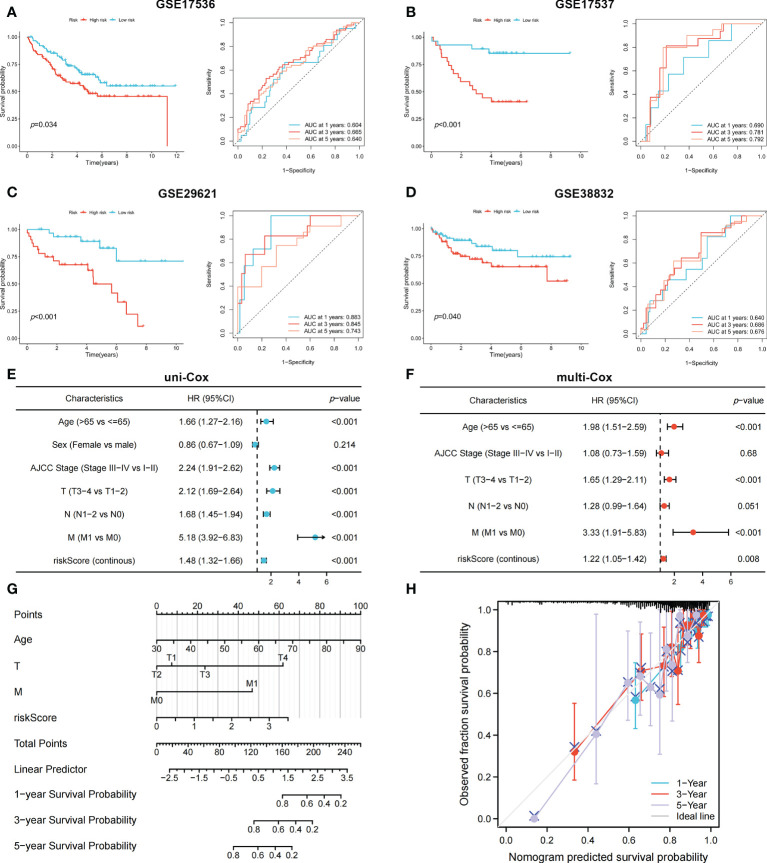
The KM and ROC methods were used to evaluate the efficiency of the risk score at predicting patient survival and construction of the nomogram model. **(A–D)** KM and ROC curves showing the prognostic value in multiple cohorts. Forest plots of univariate **(E)** and multivariate **(F)** Cox regression analyses in patients with CRC. **(G)** Construction of the nomogram model using risk score and other clinical features. **(H)** Calibration plot showing the differences between nomogram-predicted survival rates and actual survival rates.

To identify genes that could be used in the prognostic signature, DEGs between OFRG clusters were identified. Among these genes, *S100A8*, *TRIB2*, *PLA2G2A*, *RGS2*, *CCL8*, *CXCL13*, *CXCL9*, *RAMP1*, *SFRP4*, *C10orf99*, and *OLFM4* were screened as PRDEGs using univariate Cox regression analysis (**Figure 3A**, *p* < 0.05). *CXCL9*, *CXCL13*, *CCL8*, *PLA2G2A*, and *TRIB2* were identified as signature genes using LASSO (**Figures 3B, C**) and the stepwise Cox regression method, and the coefficient values are shown in **Figure 3D** and **Supplementary Table S4**. The risk score was calculated according to expression levels of five signature genes and corresponding coefficient values using the formula below: risk score = [expression level of *CXCL9* × (-0.145099)] + [expression level of *CXCL13* × (-0.130486)] + [expression level of *CCL8* × (0.230145)] + [expression level of *PLA2G2A* × (-0.072124)] + [expression level of *TRIB2* × (0.297347)]. Patients with CRC were divided into high- and low-risk groups based on the risk score, and high-risk patients had a higher risk of mortality (**Figure 3E**). The KM curve also showed that high-risk patients had significantly worse prognosis than low-risk patients (*p* < 0.001, **Figure 3F**). The AUC values of 1-, 3-, and 5-year survival were 0.665, 0.652, and 0.637, respectively (**Figure 3G**). Pie charts showed that high-risk patients were more likely to have CRC with more advanced pathological stage than low-risk patients (**Figure 3H**). To further evaluate the efficiency of the risk score in predicting patient survival, KM and ROC analyses were performed on four independent CRC cohorts, which were GSE17536 (**Figure 4A**, *p* = 0.034, 1-year AUC = 0.604, 3-year AUC = 0.665, 5-year AUC = 0.640), GSE17537 (**Figure 4B**, *p* < 0.001, 1-year AUC = 0.690, 3-year AUC = 0.781, 5-year AUC = 0.792), GSE29621 (**Figure 4C**, *p* < 0.001, 1-year AUC = 0.883, 3-year AUC = 0.845, 5-year AUC = 0.743), and GSE38832 (**Figure 4D**, *p* = 0.040, 1-year AUC = 0.640, 3-year AUC = 0.686, 5-year AUC = 0.676). Factors with a *p*-value <0.05 in the univariate analysis (**Figure 4E**) were included in the multivariate analysis (**Figure 4F**). Age (*p* < 0.001, HR = 1.98, 95% CI [1.51–2.59]), T (*p* < 0.001, HR = 1.65, 95% CI [1.29–2.11]), M (*p* < 0.001, HR = 3.33, 95% CI [1.91–5.83]), and risk score (*p* = 0.008, HR = 1.22, 95% CI [1.05–1.42]) remained significant after multivariate analysis, and these four factors were included in the nomogram model (**Figure 4G**). The calibration graph showed that the nomogram-predicted survival rates were close to actual the survival rates (**Figure 4H**).

### Immune cell infiltration differences in high- and low-risk groups

**Figure 5:**
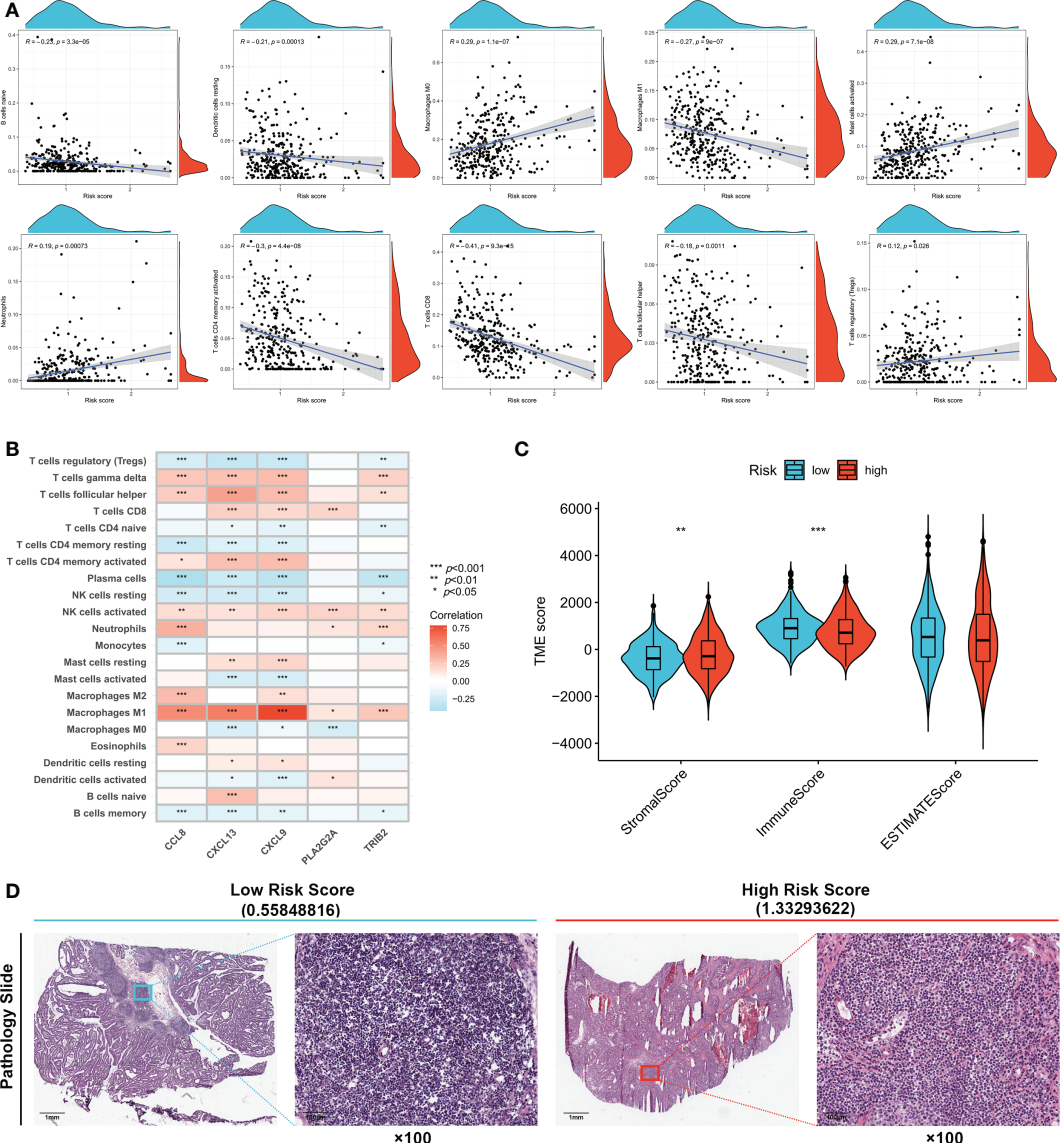
Evaluation of tumor microenvironment in high- and low- risk groups. **(A)** Relationship between risk score and different immune cell types. **(B)** Correlation between the abundance of immune cells and five genes in the prognostic signature. **(C)** Correlation between risk score and immune-related scores. **(D)** TCGA Pathology Slides confirmed that immune cell infiltration was greater in the tumor of low-risk patients than in high-risk patients. *p < 0.05; **p < 0.01; and ***p < 0.001.

The correlation between immune cell infiltration and the risk score is presented in **Figure 5A**. Ten types of immune cells were correlated with the risk score (*p* < 0.05). The association between the risk score and five signature genes is shown in **Figure 5B**. Stromal, immune, and ESTIMATE scores were also compared between high- and low-risk groups. **Figure 5C** shows that the low-risk group had a lower stromal score (*p* < 0.01) and higher immune score (*p* < 0.001), indicating increased immune cell infiltration levels in the low-risk group. TCGA pathology slides confirmed that immune cell infiltration was greater in the tumors of low-risk patients than in those of high-risk patients (**Figure 5D**).

### Relationship between risk score and IC_50_ values of therapeutic drugs

**Figure 6:**
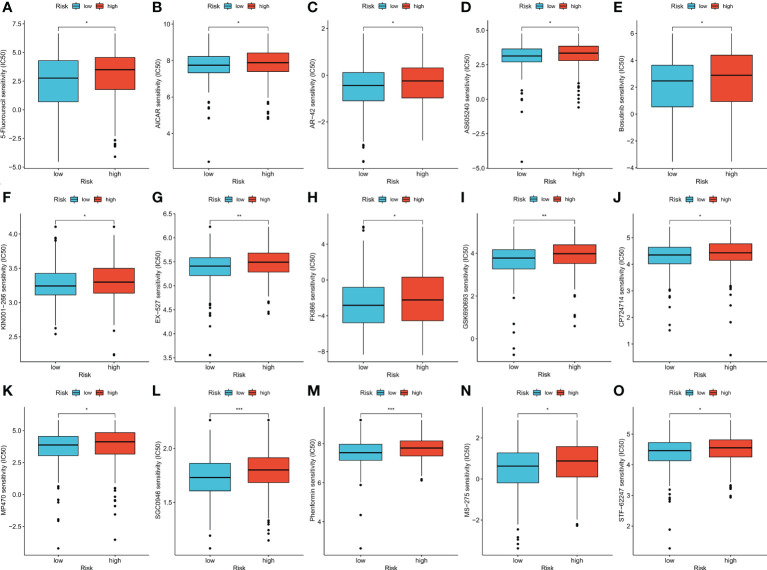
**(A–O)** Therapeutic drugs showed significant IC_50_ differences in high- and low-risk groups. *p < 0.05; **p < 0.01; and ***p < 0.001.

The therapeutic effects of 15 types of drug molecules were evaluated using IC_50_ values (**Figure 6A**). The IC_50_ of 5-fluorouracil was significantly higher in the high-risk group (*p* < 0.05), indicating that low-risk patients might have better responses to fluorouracil-based chemotherapy. Low-risk patients also had higher sensitivities to the other 14 types of drug molecules (**Figures 6B–O**, *p* < 0.05).

### Efficiency of risk score in predicting patient response to ACT and bevacizumab

**Figure 7:**
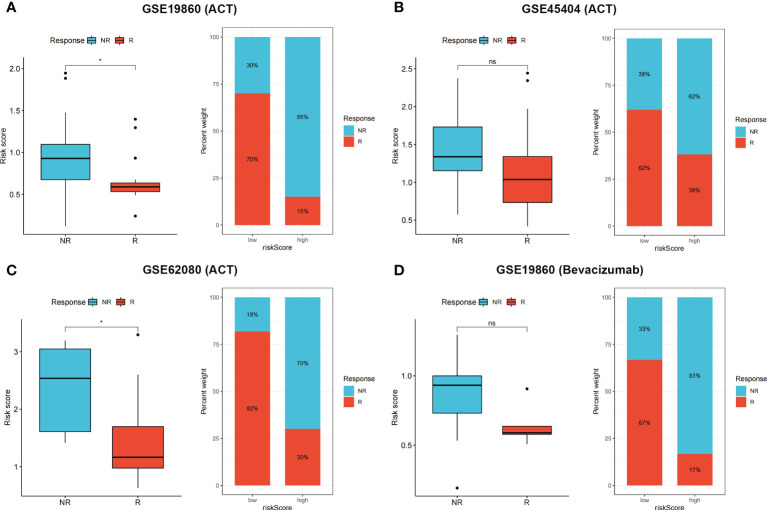
**(A–D)** Non-responders to 5-Fluorouracil chemotherapy and bevacizumab had higher risk score in multiple cohorts, the proportion of NR and R patients in these three cohorts was also shown. ns p > 0.05 and *p < 0.05.

To validate patient responses to drug therapy, three independent CRC cohorts, including transcriptional data and the complete information of patients’ responses to drug therapy, were used. Patients with no response to 5-fluorouracil chemotherapy had higher risk scores in GSE19860 (**Figure 7A**, *p* < 0.05), GSE45404 (**Figure 7B**, *p* > 0.05), and GSE62080 (**Figure 7C**, *p* < 0.05) cohorts, and the proportions of patients in NR and R groups among these three cohorts are also shown. In the GSE19860 cohort, 12 patients also received bevacizumab therapy, and non-responders exhibited higher risk scores than responders (*p* > 0.05). Specifically, 67% of patients in the low-risk group were responders to bevacizumab, whereas only 17% patients were responders to bevacizumab in the high-risk group (**Figure 7D**).

### Immune checkpoints expression, TIDE score, and IPS in the high- and low-risk groups

**Figure 8:**
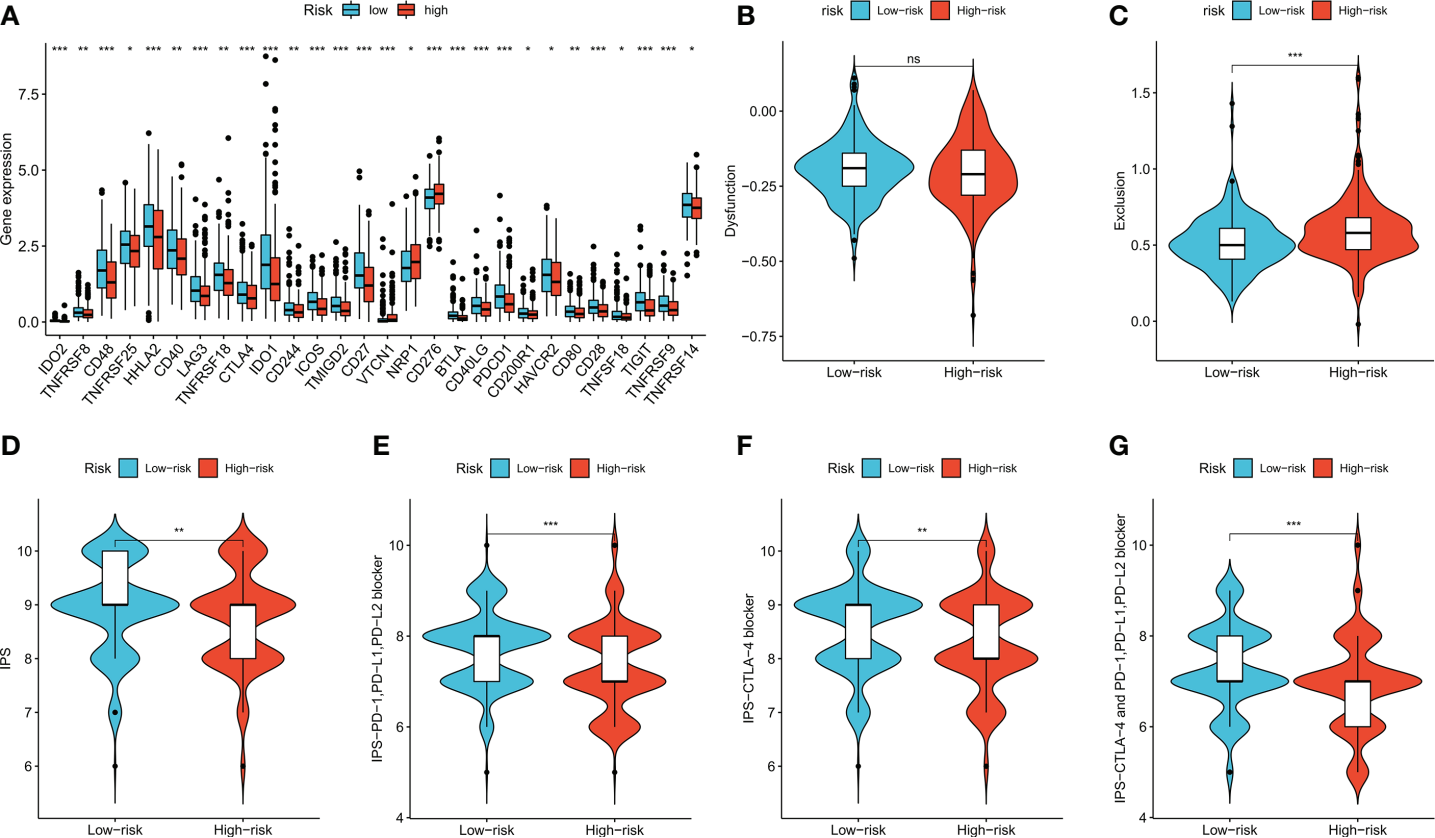
Immune checkpoint genes expression, TIDE score and IPS of patients in two risk groups. **(A)** The differences of immune checkpoint gene expression in high-risk and low-risk groups. **(B, C)** Violin plots showed the relationship between TIDE score and risk groups. **(D–G)** Violin plots showed the relationship between IPS and risk groups. *p < 0.05; **p < 0.01; and ***p < 0.001. ns p > 0.05.

Expression levels of some well-known immune checkpoint genes in the high- and low-risk groups were further compared, and the results showed that the low-risk group had higher immune checkpoint expression, including *PD-1* (*PDCD1*), *LAG-3*, and *CTLA-4* (**Figure 8A**, *p* < 0.05), suggesting that low-risk patients might have better responses to immunotherapy. The TIDE score is used to predict the probability of immune escape, and immune dysfunction scores (**Figure 8B**, *p* > 0.05) were not significantly different between the low- and high-risk groups, whereas the high-risk group had higher immune exclusion scores (**Figure 8C**, *p* < 0.001), indicating a higher likelihood of immune exclusion and a worse response to immunotherapy. IPSs between the two groups were also compared, and low-risk patients who received different types of immune checkpoint blockade therapy had significantly higher scores (**Figures 8D–G**, *p* < 0.01), also suggesting that low-risk patients might have better responses to immune checkpoint blockade therapy.

### Evaluating the performance of the risk score in predicting immunotherapy benefits

**Figure 9:**
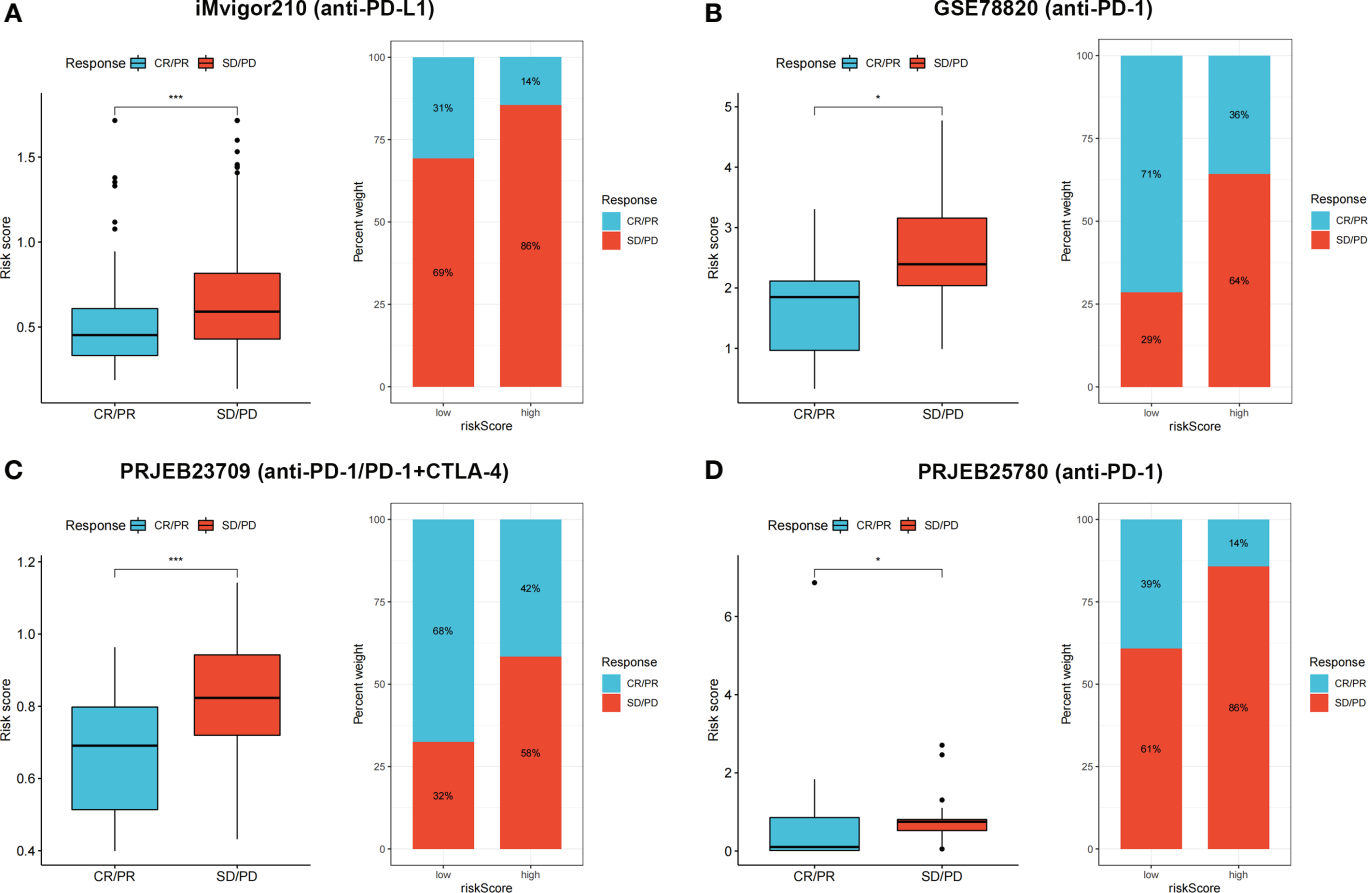
**(A–D)** CR/PR patients had lower risk score in all the four cohorts, and low risk group showed higher proportion of responders to anti-*PD-1*, anti-*PD-L1*, or combined anti-*PD-1* and anti-*CTLA-4* immunotherapy. *p < 0.05 and ***p < 0.001.

Four independent immunotherapy cohorts were applied to evaluate the performance of risk scores in predicting immunotherapy benefits. Responders had lower risk scores in all four cohorts (*p* < 0.05), and the low-risk group showed a higher proportion of responders to anti-*PD-1*, anti-*PD-L1*, or combined anti-*PD-1* and anti-*CTLA-4* therapy (**Figures 9A–D**). The results indicated that our risk score showed satisfactory performance in predicting immunotherapy benefits.

### *In vitro* verification of signature genes by qRT-PCR

**Figure 10:**
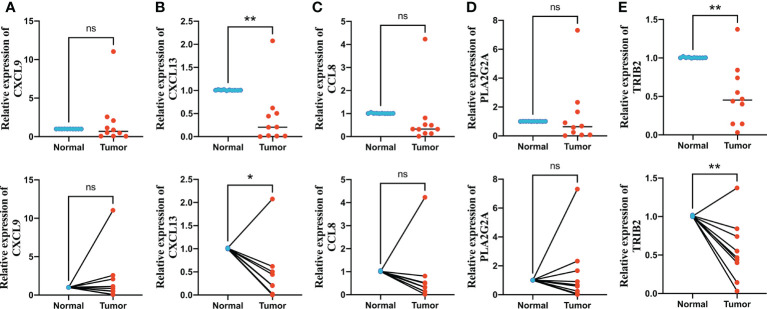
Quantitative real-time polymerase chain reaction (qRT-PCR) analyses of *CXCL9* **(A)**, *CXCL13* **(B)**, *CCL8* **(C)**, *PLA2G2A* **(D)** and *TRIB2* **(E)** expression in 10 pairs of CRC tissues and adjacent non-cancer tissues. *p < 0.05 and **p < 0.01. ns p > 0.05.

We finally performed qRT-PCR to further explore the expression levels of five signature genes in 10 pairs of human CRC and adjacent normal tissues collected after surgical resection from The First Affiliated Hospital of Anhui Medical University. Among these five genes, *CXCL9*, *CCL8*, and *PLA2G2A* did not show significant changes in expression levels between normal and tumor tissues, whereas *CXCL13* and *TRIB2* exhibited significantly decreased expression in CRC tissues compared with that in normal tissues (**Figures 10A–E**), suggesting that these two genes might be potential therapeutic targets for CRC.

## Discussion

Crosstalk between oxidative stress and ferroptosis have been identified in many human diseases. NOX4 promotes ferroptosis in astrocytes through lipid peroxidation induced by oxidative stress by impairing mitochondrial metabolism in Alzheimer’s disease (24). Oxidative stress-dependent ferroptosis can be regulated by *GDF15* post-spinal cord injury (25). It was also reported that the effects of nanomedicine in targeting ferroptosis and apoptosis can be enhanced by oxidative stress (26). In recent years, some studies focused on constructing oxidative stress or ferroptosis-related risk models for predicting patient survival and immune landscapes in various types of malignant tumors (27–31). However, few risk models were developed based on a combination of these two phenotypes.

In this study, 34 genes related to oxidative stress and ferroptosis were identified; among these OFRGs, some have been proven to be associated with development and progression of CRC. *MAPK14* is significantly related to patient survival, clinical characteristics, and immune infiltration in CRC (32). Further, *NQO1* is a biomarker for prognosis and chemosensitivity in patients with CRC liver metastasis (33). Moreover, *HSPA5* promotes CRC development by inhibiting ferroptosis through the maintenance of *GPX4* stability (34). Oxaliplatin-based chemosensitivity in CRC can be weaken by preventing PANoptosis *via* phosphorylated *NFS1* (35). Expression levels and genetic and transcriptional alterations of the 34 OFRGs were analyzed in CRC, and most of these OFRGs were differentially expressed and associated with patient prognosis. Three OFRG clusters were identified using 34 OFRGs, and patients in the three clusters showed different clinical outcomes, OFRG expression, and immune cell infiltration levels. Cluster A had an immune-desert phenotype, and it had the lowest infiltration levels of immune cells, including activated B cells, activated CD4+ T cells, eosinophils, MDSCs, macrophages, and natural killer T cells. Tumor-infiltrating immune cells can affect the response to immunotherapy, and expression levels of immune checkpoint genes can be upregulated by tumor-infiltrating CD4+ T cells (36). Eosinophils show high catalytic content of Fe (II), and the accumulation of Fe (II) can promote ROS production and eventually result in ferroptosis (37). MDSCs can inhibit functions of NK cells and T cells to promote tumor immune escape (38).

DEPRGs in the three OFRG clusters were identified and used to construct the prognostic signature. LASSO and stepwise Cox analyses were performed, and *CXCL9*, *CXCL13*, *CCL8*, *PLA2G2A*, and *TRIB2* were finally selected as signature genes to calculate the risk score. *CXCL13* correlates with poor prognosis and 5-flurourouracil resistance in CRC (39, 40). *TRIB2* acts as an oncogene in CRC by blocking cellular senescence (41). Patients with CRC were divided into high- and low-risk groups based on the calculated risk score, and low-risk patients had better prognosis than high-risk patients. Further, the efficiency for predicting prognosis was validated based on four independent CRC cohorts, suggesting that our risk score had convincing predictive ability. The risk score remained significant after univariate and multivariate Cox regression analyses, indicating that it is an independent prognostic factor for patients with CRC. In addition, a nomogram model was built based on risk scores and other clinical features; high predictive efficiency was observed based on the calibration graph. The TME consists of cellular components, including stromal cells, endothelial cells, and immune cells, and non-cellular components, including cytokines, growth factors, matrix proteins, nucleic acids, and metabolites (42). The TME plays a vital role in tumor occurrence, progression, and chemotherapy resistance (43). The risk score correlated with various types of immune cells, and four such types were positively associated with the risk score, whereas the other six types of immune cells were negatively related to the risk score. Immune-related scores can be used to predict the efficacy of chemotherapy and immunotherapy (44). In this study, the low-risk group had lower stromal scores and higher immune scores, indicating higher immune infiltration levels and better responses to chemotherapy and immunotherapy. To further verify our findings, we examined drug susceptibility, immune checkpoint expression, TIDE scores, and IPS scores in high- and low-risk groups. The low-risk group had lower IC_50_ values with respect to 15 types of therapeutic drugs, including 5-fluorouracil, suggesting that low-risk patients might be more sensitive to fluorouracil-based chemotherapy. Thus, three independent CRC cohorts were used, and the results suggested that low-risk patients have better responses to fluorouracil-based chemotherapy. It was also found that non-responders to bevacizumab had higher risk scores. Immune checkpoints were expressed at higher levels in the low-risk group, and lower exclusion scores and higher IPSs were also observed, suggesting a lower probability of immune escape and better responses to immune checkpoint blockades. These findings were verified using four immunotherapy cohorts with melanoma, urothelial, or metastatic gastric cancer. In addition, qRT-PCR was performed to explore the expression differences between CRC and adjacent normal tissues, and the results suggested that *CXCL9* and *TRIB2* might be potential diagnostic or therapeutic targets of CRC.

Clinically, tumor markers, including carcinoembryonic antigen, carbohydrate antigen 125 (*CA125*), and carbohydrate 199 (*CA199*), as well as the AJCC staging system, are widely used to evaluate tumor progression and prognosis in the peri-operation of patients with CRC. However, chemotherapy is recommended after surgery for advanced stage CRC, and tumor markers and the AJCC stage cannot be used to accurately predict therapeutic responses. To address this issue, MSI, TMB, and NAL were identified as new biomarkers. MSI is caused by different mismatch repair mechanisms, which are strongly related to the response to *PD-1* blockade therapy (45). Patients with high-MSI CRC benefit significantly less from neoadjuvant chemotherapy (46). TMB shows predictive value for non-small-cell lung cancer patients treated with *PD-1*/*PD-L1* blockade therapy (47, 48). The correlation between the NAL and immunotherapy response in solid tumors has also been clarified in previous studies (49–51). However, these biomarkers do not show perfect predictive ability because they are associated with a small percentage of the patient population or moderate efficiency. We constructed a novel oxidative stress- and ferroptosis-related gene prognostic signature, which can used to predict patient prognosis, the immune landscape, and therapeutic responses in CRC; further, the signature showed satisfactory efficiency in distinguishing cold and hot tumors.

However, there are certain limitations to this study. First, our analysis and conclusions were based on public databases and retrospectively collected tumor samples, which might cause inherent case selection bias. Although our findings were validated using multiple cohorts, clinical samples should be collected from a larger cohort of patients to further verify our conclusion. Second, our sample size for verification experiments was limited, and more in-depth *in vitro* and *in vivo* experiments are required to further explore the functions of OFRGs in CRC. Finally, clinical information related to surgery and tumor markers was not considered. Thus, more clinical cases are needed to confirm our conclusion. In conclusion, we constructed a novel prognostic signature based on OFRGs, which showed satisfactory efficiency in predicting patient prognosis, the immune landscape, and therapeutic effects in CRC.

## Data availability statement

Publicly available datasets were analyzed in this study. The names of the repositories and accession numbers are contained within the article/**Supplementary Materials**.

## Ethics statement

The studies involving human participants were reviewed and approved by Ethics Committee of The First Affiliated Hospital of Anhui Medical University. The patients/participants provided their written informed consent to participate in this study.

## Author contributions

XW, YX and LD are responsible for writing and submitting the manuscript. ZY, MW, SC, RS, and QH are responsible for data collection and analysis. JC, XZ, ZW and XH are responsible for the production of pictures. YY and HZ are responsible for final check of the manuscript. KH, HBZ and WC are responsible for the ideas and guidance. All authors contributed to the article and approved the submitted version.

## Funding

The work was supported by the National Natural Science Foundation of China (No.81670517 and 81870402) and Research Fund of Anhui Institute of Translational Medicine (2021zhyx-C30).

## Acknowledgments

We acknowledged TCGA and GEO database for providing their platform and contributors for uploading their meaningful datasets.

## Conflict of interest

The authors declare that the research was conducted in the absence of any commercial or financial relationships that could be construed as a potential conflict of interest.

## Publisher’s note

All claims expressed in this article are solely those of the authors and do not necessarily represent those of their affiliated organizations, or those of the publisher, the editors and the reviewers. Any product that may be evaluated in this article, or claim that may be made by its manufacturer, is not guaranteed or endorsed by the publisher.

## Supplementary material

Supplementary Table 1 Primer sequences for PCR amplification.

Supplementary Table 2 Clinical information of patients from all the cohorts in this study.

Supplementary Table 3 Thirty-four OFRGs were screened out after taking insertion between ORGs and FRGs.

Supplementary Table 4 Coefficient values of 5 signature genes in the multivariate Cox regression analysis.
