# Supplementary material for: A novel oxidative stress- and ferroptosis-related gene prognostic signature for distinguishing cold and hot tumors in colorectal cancer
Source: Front Immunol. 2022 Oct 31;13:1043738. doi: 10.3389/fimmu.2022.1043738 (PMC9660228; doi:10.3389/fimmu.2022.1043738)
Supplement: Supplementary file 5 [file DataSheet_1.docx]

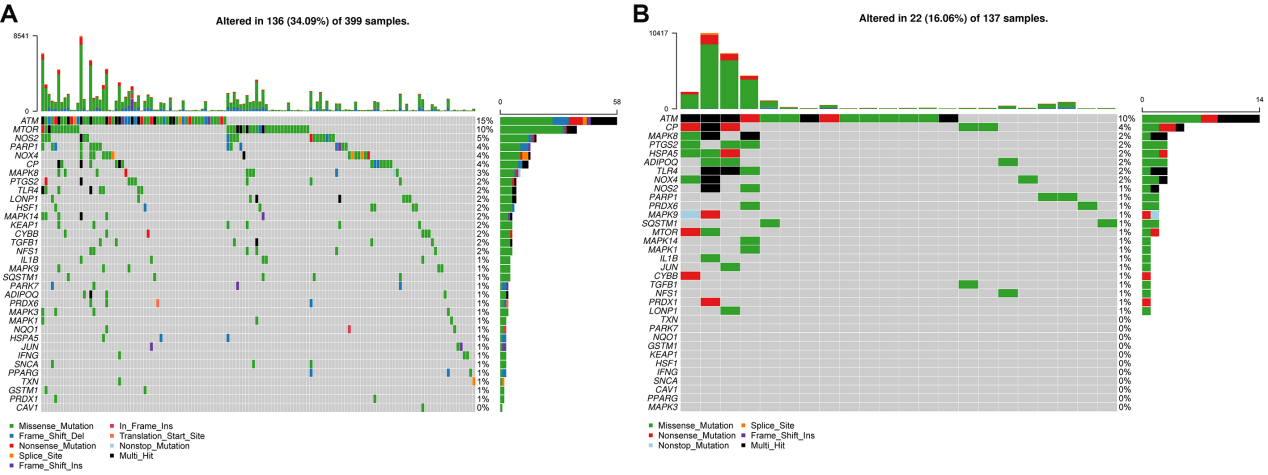


**Supplementary Figure S1:** The somatic mutation incidence of OFRGs in patients with COAD (A) and READ (B). COAD: colon adenocarcinoma; READ: rectum adenocarcinoma.


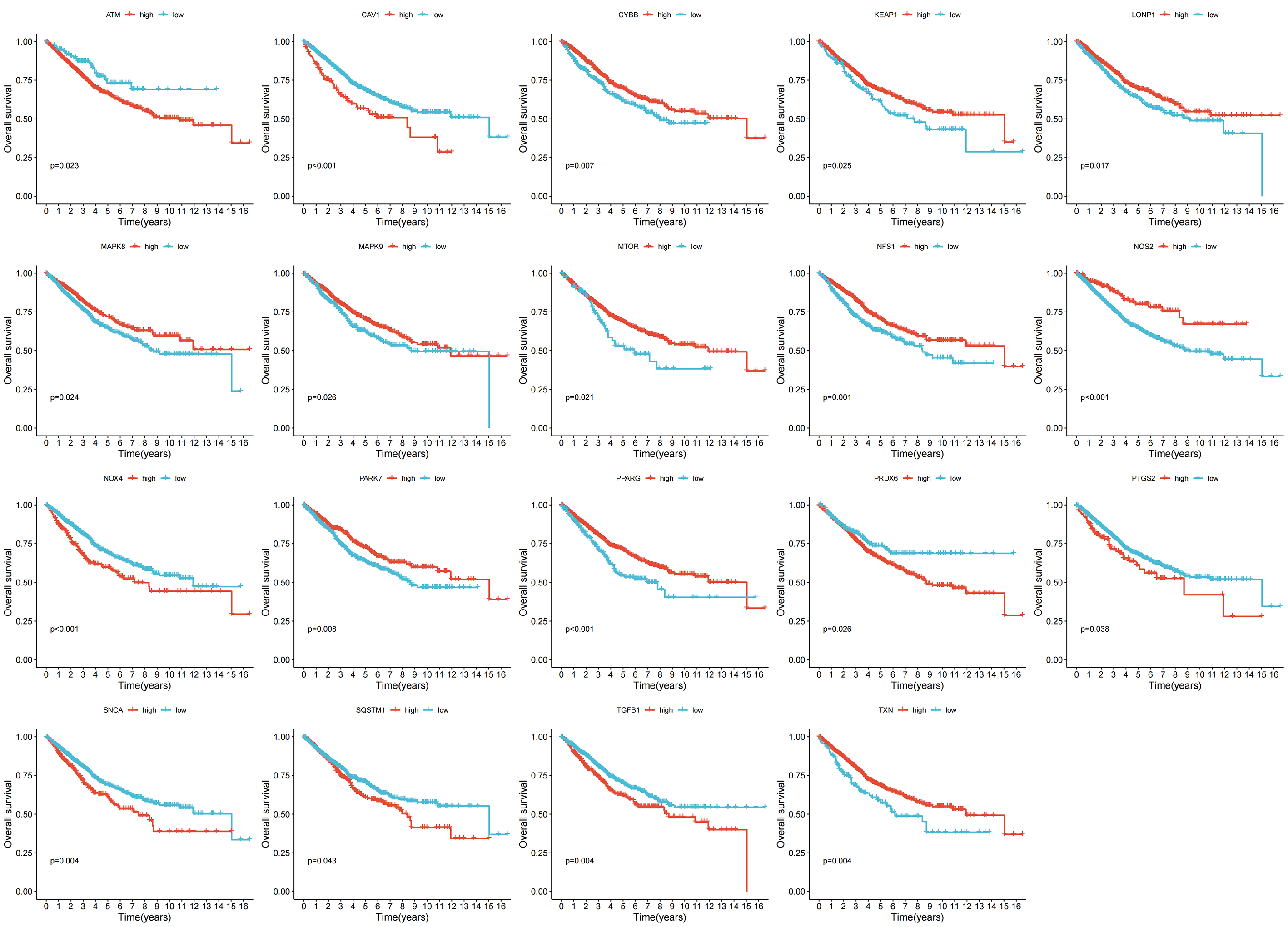


**Supplementary Figure S2:** Survival curves of prognosis-related OFRGs.


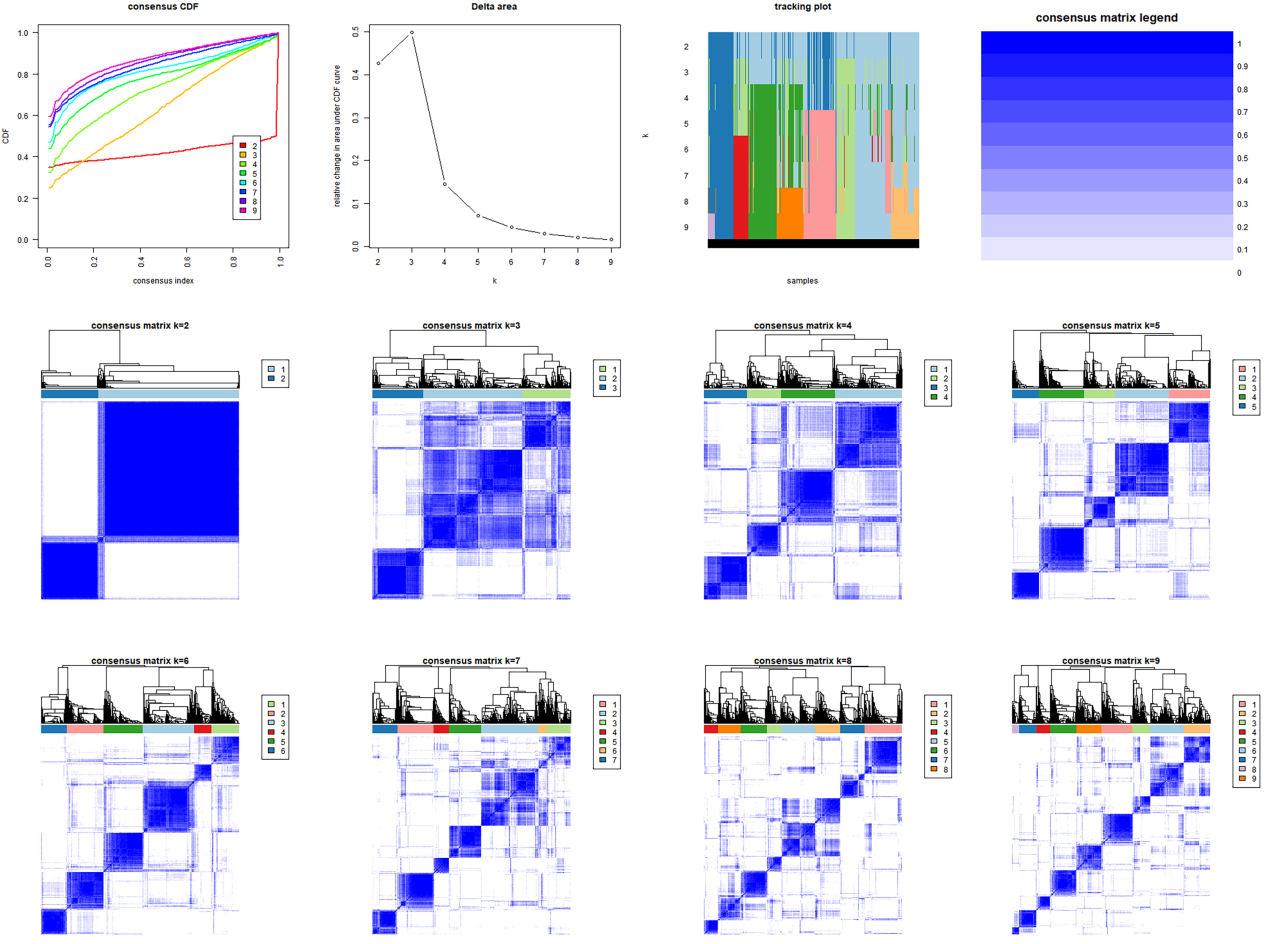


**Supplementary Figure S3:** The expression of the TRGs were used to perform a consensus clustering analysis to classify patients into two TRG clusters.


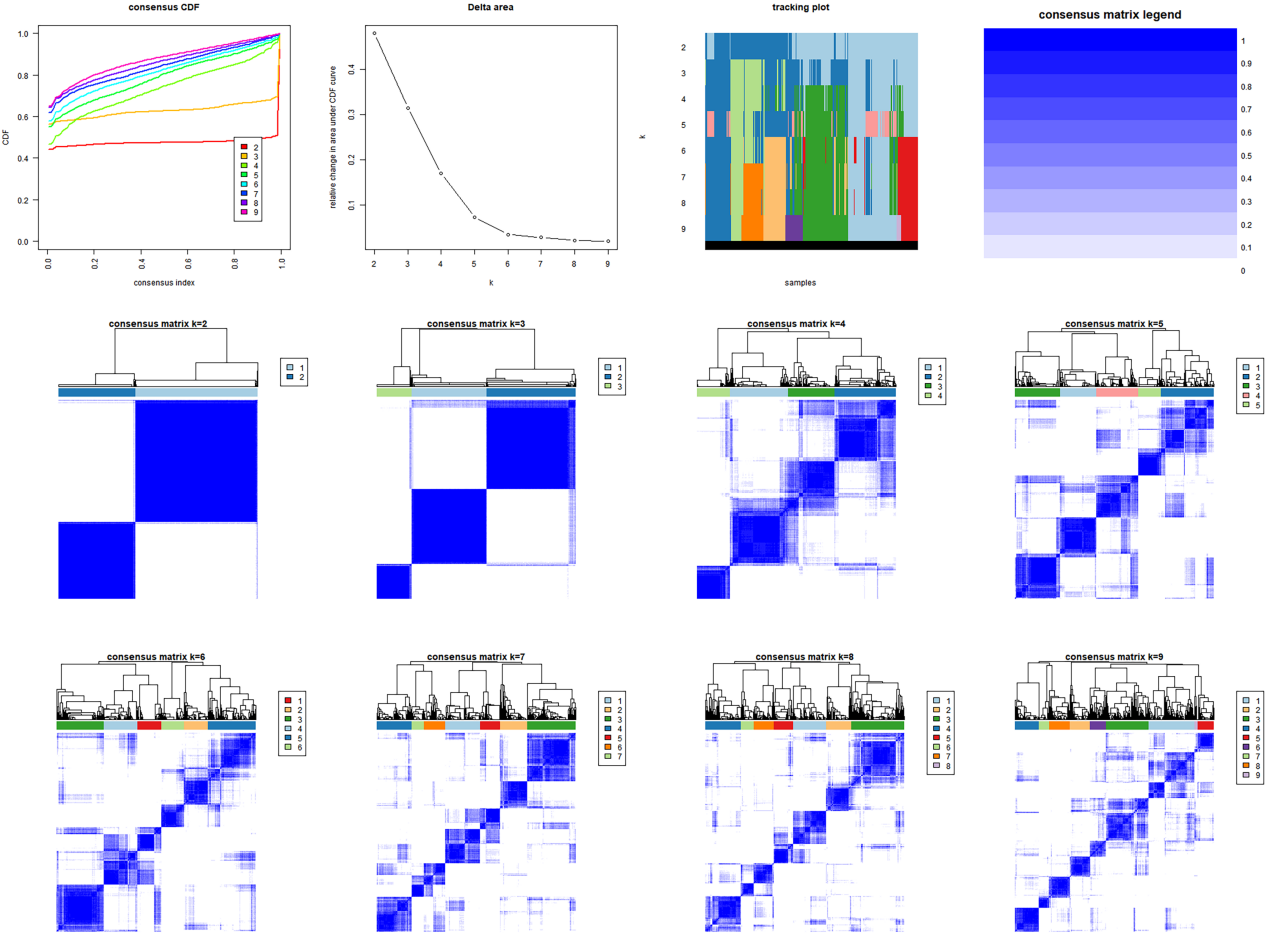


**Supplementary Figure S4:** Patients with CRC were divided into three gene clusters using consensus clustering.
